# A spiral cystic fibroma originating from left ventricular fascicular muscle: a case report

**DOI:** 10.1186/s12893-022-01616-w

**Published:** 2022-05-10

**Authors:** Chong Luo, Zhong Wu, Lijie Jiang, Weitao Liang

**Affiliations:** Department of Cardiovascular Surgery, West China Hospital, Sichuan University, Guoxuexiang 37th, Chengdu, 610041 Sichuan People’s Republic of China

**Keywords:** Cardiac fibroma, Left ventricular, Adults, Tumor morphology, Surgical treatment

## Abstract

**Background:**

In adults, cardiac fibromas are fairly rare, mostly round in shape, and few cases of ventricular fibromas of other morphology have been reported.

**Case presentation:**

We report a case of a 47-year-old male patient admitted with recurrent nocturnal paroxysmal dyspnea, diagnosed by transthoracic cardiac ultrasound, transesophageal ultrasound, and computed tomography (CT) as a left ventricular occupancy with a spiral shape resembling a conch with a fixed base and a free distal end.

**Conclusion:**

This case reports a rare but noteworthy morphological features of the adult uncommon ventricular tumor pathological type. Furthermore, the patient had no notable postoperative issues and was followed up on for a year following surgery, with no residual tumors or arrhythmias discovered during the examination.

**Supplementary Information:**

The online version contains supplementary material available at 10.1186/s12893-022-01616-w.

## Background

The incidence of primary cardiac tumors is extremely low, ranging from 0.0017 to 0.00195 according to previous literature [1]. Cardiac primary fibromas are quite uncommon in adults. The disease manifestation is not specific or may be asymptomatic. However cardiac fibromas are mostly round in shape [2–4], few cases of other morphology have been reported.

## Case presentation

A 47 year-old male patient with recurrent nocturnal paroxysmal dyspnea was admitted to the local hospital. The patient was diagnosed as primary cardiac tumor, and then transferred to the West China Hospital for surgical treatment.

**Fig. 1:**
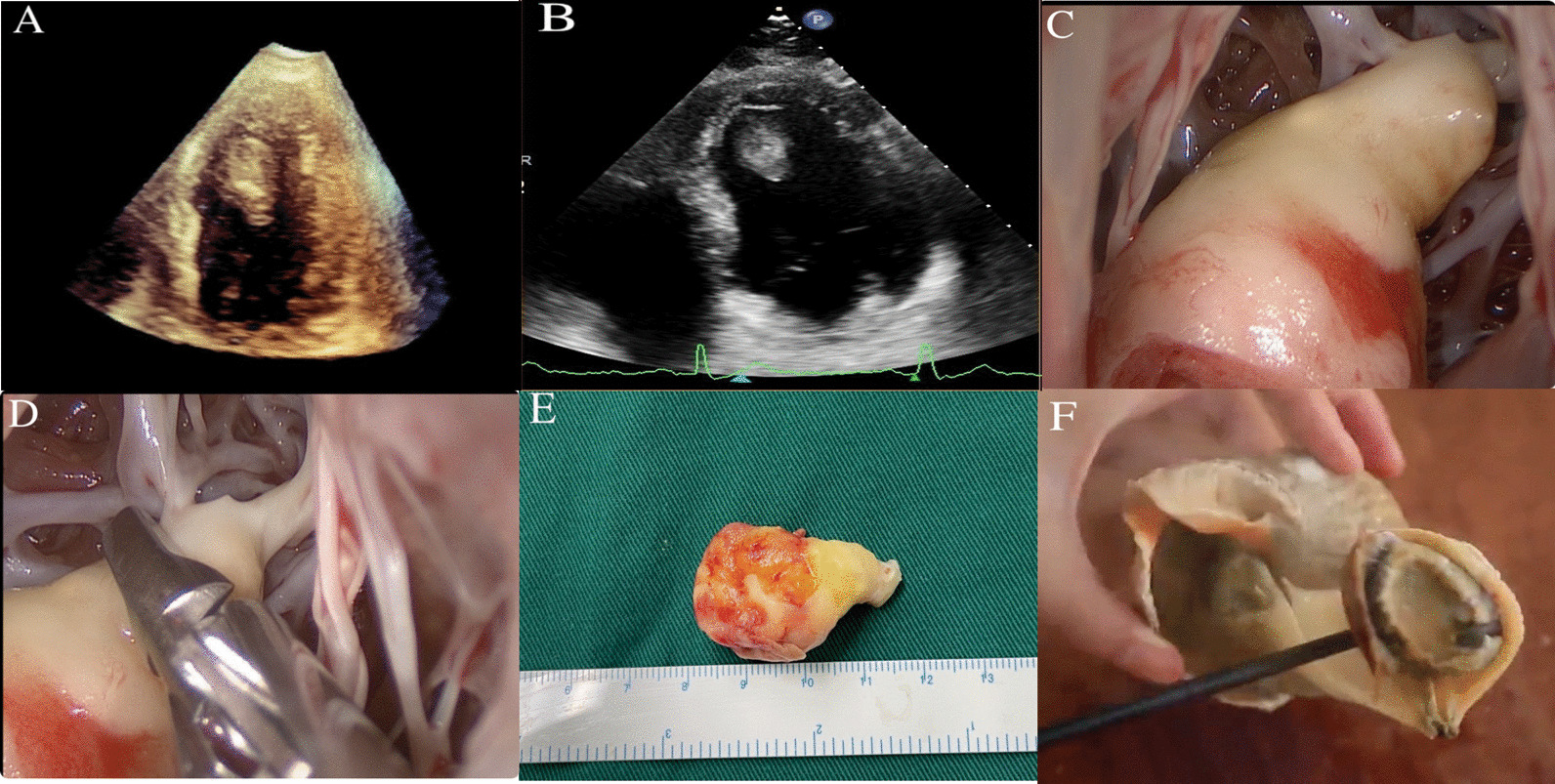
**A**, **B** Transthoracic echocardiography revealed that a spiral cystic mass, 27 × 14 mm, located on the left ventricular apex. It looked like a conch floating in the left ventricle. **C**, **D** Intraoperative findings: A spiral cystic shape and 1.5 * 5 cm size of mass rooted on the left ventricular apex. The tumor was mainly soft and fragile, while the basement was partly calcified. **E**, **F** The conch has a similar silhouette and is an appropriate eponym of this sign

Transthoracic echocardiography (Fig. 1A, B; Additional file 1: Movie S1 and Additional file 2: Movie S2) and transesophageal echocardiography revealed that a spiral cystic mass, 27 × 14 mm, located on the left ventricular apex. It looked like a conch floating in the left ventricle.

The operation was performed under standard cardiopulmonary bypass. It was performed through the traditional atrial septal incision under thoracoscopic assistance. Intraoperative findings: (Fig. 1C, D). A spiral cystic shape and 1.5 * 5 cm size of mass rooted on the left ventricular apex. The tumor was mainly soft and fragile, while the basement was partly calcified. The tumor’s morphology (Fig. 1E, F) has a similar silhouette to the conch. The tumor mass was resected completely, and the histopathological examination confirmed a fibroma. Postoperative transesophageal echocardiography showed that the functions of left ventricles were well preserved. The patient had a good postoperative recovery and was discharged on day 9 post-surgery.

## Discussion and conclusion

The incidence of primary cardiac tumors is extremely low, ranging from 0.0017 to 0.00195 according to previous literature [1]. Cardiac primary fibromas, the second most common benign cardiac tumor after rhabdomyosarcoma in the pediatric population, are quite uncommon in adults. It originates from fibroblasts present in various parts of the heart (mainly found in the ventricles or septum). Although cardiac fibromas are benign tumors, their clinical manifestations are related to the site and size of growth because the tumor grows inside the heart chambers, and the main symptoms include symptoms such as chest pain, heart enlargement, arrhythmias, and even sudden death, or may be asymptomatic [5–7]. And arrhythmias are very common in such tumors occurring in the free ventricular wall or septal area, and ST-T wave abnormalities are always present [8] and as the size of the tumor increases, the risk of necrosis causing by inadequate blood supply to the systematic circulation, mitral valve obstruction increases accordingly. The case in this study is a 47-year-old male admitted with recurrent nocturnal paroxysmal dyspnea, diagnosed as left ventricular occupancy by transthoracic cardiac ultrasound, transesophageal ultrasound, and CT, Cardiac ultrasound indicated that the tumor was a narrow basal morphology and the free portion seemed to float spirally in the left ventricular cavity. Intraoperatively, the tumor was stretched by forceps, resembling a spinal conch. And the histopathological examination confirmed a fibroma. Postoperative transesophageal echocardiography showed that the functions of left ventricles were well preserved. The patient had a good postoperative recovery and was discharged on day 9 post-surgery. Furthermore, the patient had no notable postoperative issues and was followed up on for a year following surgery, with no residual tumors or arrhythmias discovered during the examination.

From reviewing previous case reports of ventricular fibroma, we found that most cardiac fibromas grow in a round-like shape into the cavity and occupy the cavity volume [2–4, 9, 10], the fibroma reported here has a special morphology with a narrow base and the body extending into the left ventricular cavity, which will not only occupy the cavity volume and cause embolism in the systematic circulation by necrosis, but also oscillate in the cavity with the contraction and diastole of the ventricle, it may repeatedly rub against the mitral valve and cause mitral valve fibrosis or embolism, leading to mitral valve stenosis, obstruction or systemic embolism.

The case reported here further demonstrates the importance of preoperative imaging image evaluation, which plays an important role in determining the morphology and location of the tumor with possible complications and assessing the patient’s prognosis. CT can present the extent of tumor infiltration in cross-sectional form as well as the location and relationship to the normal myocardium, further allowing for preoperative assessment of the extent of surgical resection and whether damage to the conduction tract will occur. Magnetic resonance imaging (MRI) itself provides a better assessment of soft tissue lesions, and cardiac MRI allows analysis of peri-tumor hemodynamic changes, which can be visualized more precisely than transthoracic cardiac ultrasound, and can show the extent of tumor growth into the myocardium and the relationship of surrounding adjacent structures more clearly than CT [11].

It is worth noting that the growth of cardiac tumors will occupy the volume of cardiac cavity, compress or wrap the conduction bundle, or cause systemic embolism due to tumor necrosis or thrombosis, Meanwhile, because primary cardiac tumors are more frequent in fetal and neonatal and have a greater fatality rate than adults, surgery should be the first option for older patients [12–14]. Padalino et al. mentioned that cardiac fibromas do not shrink with drug therapy and usually require surgical resection, the above conclusions are also acquired after reviewing previously published articles by Nwachukwu et al. [15, 16] We suggest that a preoperative multi-disciplinary treatment evaluation plays a considerable role in the perioperative period and in the development of the surgical plan.

## Supplementary Information

**Additional file 1:** Dynamic images of transthoracic echocardiography movie S1.

**Additional file 2:** Dynamic images of transthoracic echocardiography movie S2.

## Data Availability

Not applicable.

## Abbreviations

CT: Computed tomography
MRI: Magnetic resonance imaging
